# Pulsed-field gel electrophoresis and multi locus sequence typing for characterizing genotype variability of *Yersinia ruckeri* isolated from farmed fish in France

**DOI:** 10.1186/s13567-015-0200-5

**Published:** 2015-06-23

**Authors:** Ségolène Calvez, Catherine Fournel, Diane-Gaëlle Douet, Patrick Daniel

**Affiliations:** LUNAM Université, Oniris, École nationale vétérinaire, agroalimentaire et de l’alimentation Nantes-Atlantique, UMR1300 Biologie, Épidémiologie et Analyse de Risque en santé animale, F-44307 Nantes, France; INRA, UMR1300 BioEpAR, F-44307 Nantes, France; GDSAA, Groupe de Défense Sanitaire Aquacole Aquitain, F-40000 Mont de Marsan, France; Laboratoire des Pyrénées et des Landes, F-40000 Mont de Marsan, France

## Abstract

*Yersinia ruckeri* is a pathogen that has an impact on aquaculture worldwide. The disease caused by this bacterial species, yersiniosis or redmouth disease, generates substantial economic losses due to the associated mortality and veterinary costs. For predicting outbreaks and improving control strategies, it is important to characterize the population structure of the bacteria. The phenotypic and genetic homogeneities described previously indicate a clonal population structure as observed in other fish bacteria. In this study, the pulsed-field gel electrophoresis (PFGE) and multi locus sequence typing (MLST) methods were used to describe a population of isolates from outbreaks on French fish farms. For the PFGE analysis, two enzymes (*Not*I and *Asc*I) were used separately and together. Results from combining the enzymes showed the great homogeneity of the outbreak population with a similarity > 80.0% but a high variability within the cluster (cut-off value = 80.0%) with a total of 43 pulsotypes described and an index of diversity = 0.93. The dominant pulsotypes described with *Not*I (PtN4 and PtN7) have already been described in other European countries (Finland, Germany, Denmark, Spain and Italy). The MLST approach showed two dominant sequence types (ST31 and ST36), an epidemic structure of the French *Y. ruckeri* population and a preferentially clonal evolution for rainbow trout isolates. Our results point to multiple types of selection pressure on the *Y. ruckeri* population attributable to geographical origin, ecological niche specialization and movements of farmed fish.

## Introduction

A range of bacterial species affect aquaculture worldwide and are responsible for important economic losses as well as a substantial use of antibiotics on fish farms. *Yersinia ruckeri* is the causative bacterial agent of enteric redmouth disease that particularly affects salmonid farms (genera *Oncorhynchus* and *Salmo*) [1]. Since 1956, with the first isolation and identification of *Y. ruckeri* on a rainbow trout farm in the USA [2,3], the bacterial species has been isolated in many parts of the world: North and South America, Europe [4], Australia [5], and South Africa [6].

*Y. ruckeri* is a member of the *Enterobacteriaceae* family and is easy to identify by culture-based methods or molecular techniques such as PCR [7]. This pathogen was well described with a classification based on the O-antigens (heat-stable antigens) dividing the *Y. ruckeri* species into five (O1, O2, O5, O6 and O7) [8] or four (O1, O2, O3 and O4) [9] serogroups. Furthermore, *Y. ruckeri* can be separated in two biotypes, BT1 (motile, with phospholipase activity) and BT2 (non-motile, with no phospholipase activity) [10]. To improve our understanding of the dissemination and evolution of this species, it is necessary to analyze the population structure and evaluate its phenotypic and genetic variability.

In studies of general phenotypic and biochemical characteristics, *Y. ruckeri* has been described as highly homogeneous species [11-13]. The development of molecular typing methods has enabled an increase in accuracy in variability studies. Several different approaches have been used for typing *Y. ruckeri* strains including ribotyping [14], ERIC and REP [15,16] sequence-based PCR, pulsed-field gel electrophoresis (PFGE) [17,18] and multi locus sequence typing (MLST) [19]. In different studies, the genetic variability of *Y. ruckeri* has been described as higher [12] and as lower [17] than the phenotypic variability. Generally, the variability observed in a study depends on the pathogen, number of isolates, geographical variability of the sample and host diversity and choice of marker to assess the variability. The PFGE method was developed in 1984 [20] and has since become the gold standard [21] for identifying microbial strains and epidemiological tracing, currently being used in many international surveillance programs [22]. This method is considered to be precise, reliable and reproducible. The MLST method developed since 15 years [23] is generally performed on seven housekeeping genes. The DNA sequence analyses give information about the degree of genetic diversity and about the population structure of the bacterium of interest. This approach is considered as reproducible and easy transposable between laboratories [23,24].

The knowledge of the population structure of the bacteria is an important criterion for predicting outbreaks and designing effective disease control strategies, such as vaccination programs. The aim of our study was to describe the genetic variability of *Y. ruckeri* strains, responsable for outbreaks in rainbow trout farms in different regions of France. We used PFGE on the whole strain collection as well as MLST on a representative subset of genetic diverse strains (as revealed by PFGE) in order to compare the results of the two methods.

## Materials and methods

### Bacterial strains and culture conditions

As described previously [13], 123 isolates of *Yersinia ruckeri* were collected from rainbow trout farms in different regions of France, hereafter designed “Brittany”, “Adour Garonne” and “Other regions”. A few isolates came from other fish species, namely sturgeon (*n* = 3) and gudgeon (*n* = 1). Isolates were collected from fish with clinical signs of yersiniosis between 2005 and 2009. Some isolates came from repeated sampling from the same fish farms; in this case, there was a minimum of six months between the collection of the samples.

Eight reference strains were used: CIP 82.80^T^ (Collection de l’Institut Pasteur, Paris, France); CECT 956 (Colección Espanola de Cultivos Tipo, Valencia, Spain); NCTC 12266, NCTC 12267, NCTC 12268, NCTC 12269, and NCTC 12270 (National Collection of Type Cultures, London, UK); and TUTI EX5 (UK). All strains were grown on Tryptocase Soy Agar (TSA; Oxoid, Basingstoke, England) at 22 °C for 48 h. Strains were stored at −80 °C in Tryptocase Soy Broth (TSB; Oxoid) supplemented with 20.0% of glycerol.

Prior to the study, all isolates were confirmed as *Y. ruckeri* using a species specific PCR test and their serotype and biotype were determined [13].

### Pulsed-field gel electrophoresis

Genomic DNA from *Y. ruckeri* isolates was prepared in agarose plugs from pure cultures. Briefly, strains were grown for 48 h at 22 °C on TSA plates. Colonies were resuspended in cell suspension buffer (Tris 100 mM, EDTA 100 mM, pH 8.0) to obtain an optical density at 625 nm of 1.4–1.8. A total of 20 μL of proteinase K (20 mg mL^−1^; BioSolve) was added to 400 μL of bacterial suspension, which was then mixed with 400 μL of 2.0% agarose (Pulsed Field Certified Agarose, Bio-Rad) in TE buffer (Tris 10 mM, EDTA 1 mM, pH 8.0) and 1.0% SDS (Sodium Dodecyl Sulfate; Eurobio) at 55 °C. Subsequently, 100 μL of mix was dispensed into plug moulds. After solidification, agarose plugs were treated with lysis buffer (Tris 50 mM, EDTA 50 mM, 1.0% sarkosyl, pH 8.0) and 25 μL of proteinase K for 3 h at 37 °C with shaking. The plugs were washed twice in sterile water for 15 min at 50 °C and four times in TE buffer for 15 min at 50 °C. Plugs were stored in TE buffer at 4 °C. Each plug was divided, one part being digested with 40 U of *Not*I (New England Biolabs) and the other part with 40 U of *Asc*I (New England Biolabs) at 37 °C overnight. Strain CIP 82.80^T^ was prepared under the same conditions for each gel and used as a control. The migration was performed using a 1.3% agarose gel in 0.5X Tris-Borate-EDTA buffer (10X) at 14 °C in a ChefDR III system (Bio-Rad) with an electric field of 6.0 V cm^−1^ and angle of 120°. Pulse times were 1 to 16 s for 22 h then 2.5 to 8 s for 3 h for gel with plugs digested by *Not*I, and 1 to 16 s for 26 h for gel with plugs digested by *Asc*I. A standard molecular weight marker, MidRange PFG Markers II (New England Biolabs), was included three times on each gel. Gels were stained with ethidium bromide and visualized on an UV transilluminator.

### Genetic analyses

The genetic analyses were performed using the BioNumerics software (version 6.5; Applied Maths). Each gel was checked by the densitometric curve for presence or absence of bands. The bands of between 24 and 242 Kb were included in the analysis. For each experiment (*Not*I and *Asc*I enzymes), dendrograms of isolates were generated using an unweighted pair group method with arithmetic mean (UPGMA) approach and the Dice similarity coefficient, with a band optimization of 1.0% and band position tolerance of 2.0%. Means from each experiment were used to construct the similarity matrices.

According to the guidelines for interpreting chromosomal DNA restriction patterns produced by PFGE [25], patterns with ≥80.0% similarity (fewer than six bands of difference) were considered as the same cluster with closely or possibly related isolates and patterns with <80.0% similarity (six or more bands of difference) represented different clusters with unrelated isolates. Subclusters (Sc) were defined for a similarity ≥90.0%.

Diversity values were based on Simpson’s original index (Σ pi^2^), assessed as described by Hunter and Gaston [26], D = 1-Σ pi^2^. Simpson’s original index is a measure that evaluates the probability that two entities taken at random from the sample represent the same type. Conversely, the index of diversity is a measure that evaluates the probability that two entries represent different types.

### Multi locus sequence typing

DNA was extracted using the DNA extraction Kit of Promega (USA) and stored at −20 °C. The MLST scheme used was described by Bastardo et al. [19] with some modifications for amplification conditions. Briefly, six housekeeping genes, *glnA*, *gyrB*, *dnaJ*, *thrA*, *HSP60* and *recA* were amplified with primers previously described [27-29], except for *thrA* gene for which primers thrA_F: 5′ TGCTCCCATCCACAGTGC 3′ and thrA_R: 5′ GTCATCACAAATCCTGCC 3′ were used. The PCR conditions were as follows: 94 °C for 5 min, 35 cycles with 94 °C for 45 s, 62 °C for 45 s and 72 °C for 1 min, and then a final elongation at 72 °C for 5 min. The annealing temperature used was 65 °C and 51 °C for *gyrB* and *dnaJ* respectively. The PCR products were sequenced in both directions (GATC Biotech, Cologne). The quality of chromatograms was checked visually and sequences were assembled with the BioEdit 7.2 software. Numbers were assigned to the allele types (AT) manually and incremented whenever the nucleotide sequence was different compared with sequences deposited at the PubMLST site at Oxford University [30]. The combination of the AT of the six loci defined the sequence type (ST) of each isolate.

### Population genetic analysis

The following analyses were performed on all isolates and all loci using the START2 software [31]: average G + C content, allele frequency, number of polymorphic sites, pairwise ratio of non-synonymous to synonymous substitutions (dN/dS) and index of association (I^S^_A_). The nucleotide diversity per site (Pi) and the gene diversity index (H) were calculated for all isolates analysed by MLST by DnaSP 5.10 software and using LIAN 3.6 respectively.

The I^S^_A_ calculation reports the linkage disequilibrium between alleles and by extension gives an indication of the recombination events. If I^S^_A_ = 0, the loci are at equilibrium, with a random association of alleles, so recombination events are responsible for the diversity of the population structure (= pancmitic population). Conversely, if I^S^_A_ ≠ 0, the loci show a linkage disequilibrium with a preferential association of alleles, so the recombination events are absent or rare and clonal evolution is responsible for the population structure (= clonal population) [32]. The pairwise homoplasy index (PHI) and split decomposition tree obtained with Splitstree four software [33] were also indicators of recombination events.

### Phylogenetic analysis

The e-BURST V3 software was used to analyze the relation among STs and to divide them into groups clonal complexes (CC). The group parameters were assessed by bootstrap method (*n* = 1000) and the analysis was performed with default setting (a minimum of five alleles shared = SLV, single locus variant) and relaxed settings (a minimum of four alleles shared = DLV, double locus variant). A singleton is a ST not included into a CC.

### Nucleotide sequence accession numbers

The nucleotide sequences corresponding to new allele types have been deposited in the GenBank database under accession numbers KP894095 to KP894099.

## Results

### Pulsed field gel electrophoresis

**Figure 1:**
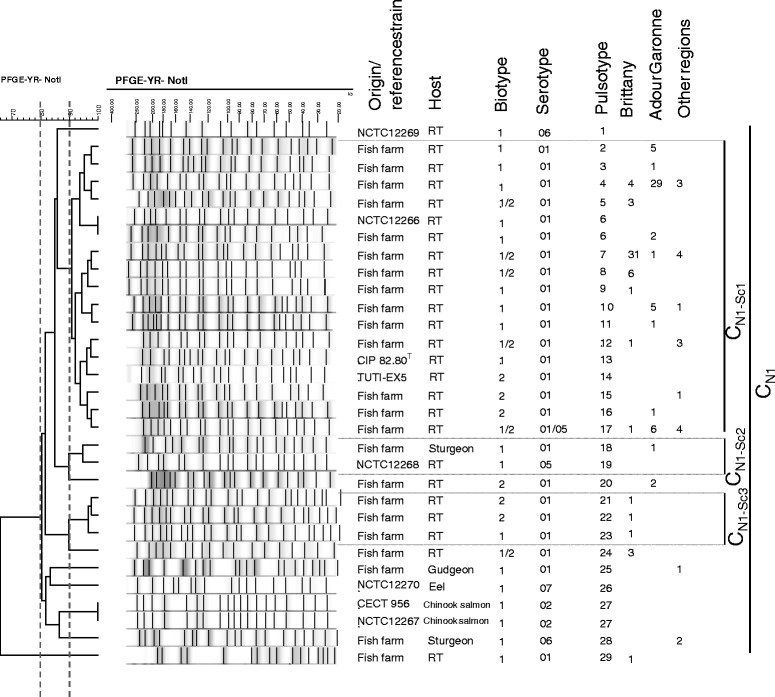
**Dendrogram of the PFGE profiles of** ***Y. ruckeri*** **(field isolates and reference strains) obtained using the** ***Not*** **I restriction enzyme and the BioNumerics software.** Origin/reference strains, host, biotype, serotype, pulsotype, number and region of origin are indicated. Clusters and subclusters are presented.

**Figure 2:**
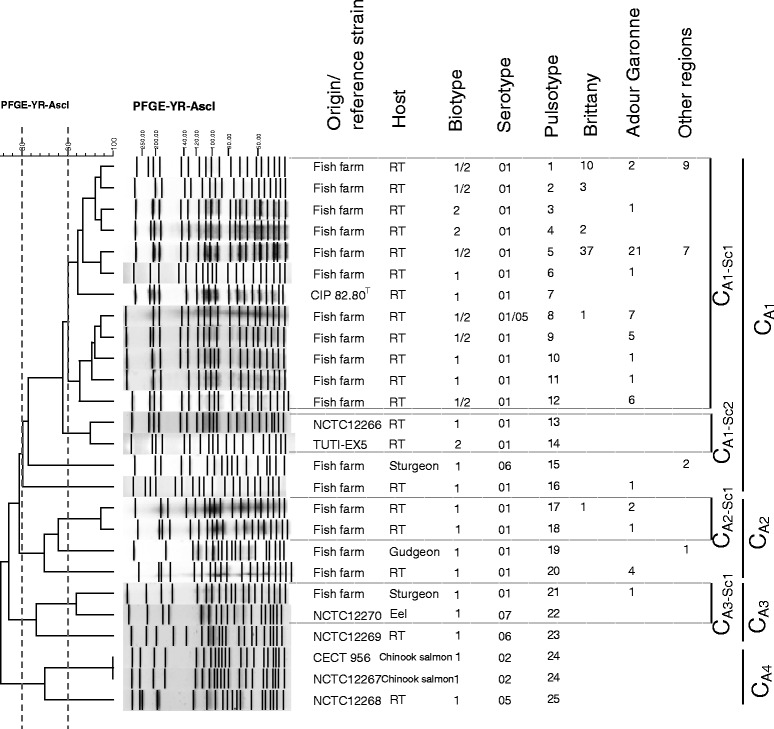
**Dendrogram of the PFGE profiles of** ***Y. ruckeri*** **(field isolates and reference strains) obtained using the** ***Asc*** **I restriction enzyme and the BioNumerics software.** Origin/reference strains, host, biotype, serotype, pulsotype, number and region of origin are indicated. Clusters and subclusters are presented.

**Figure 3:**
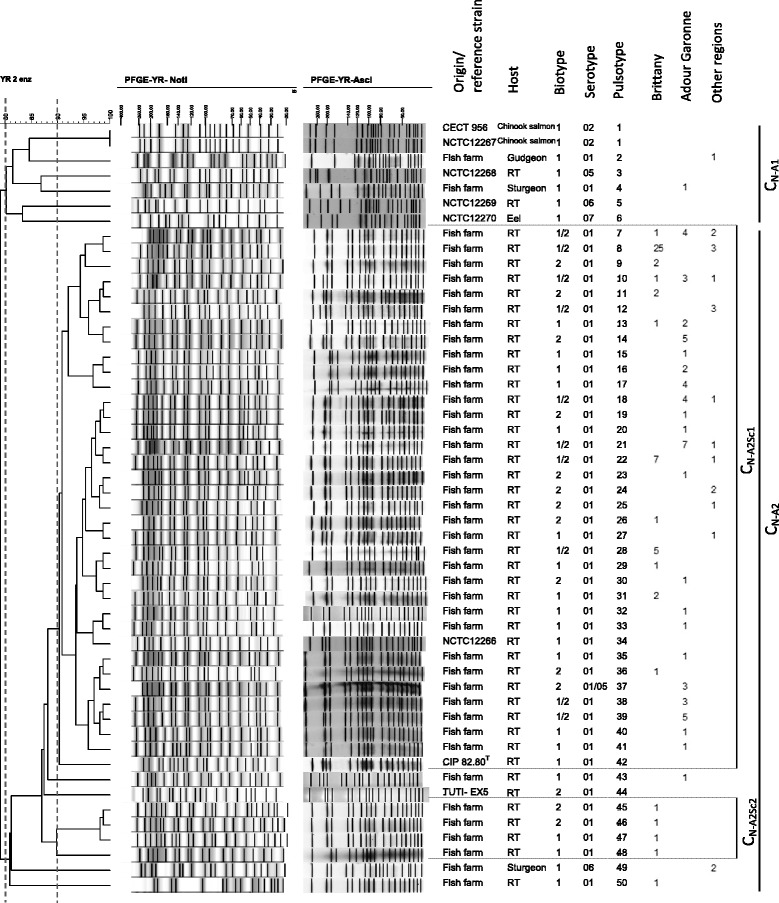
**Dendrogram of the PFGE profiles of** ***Y. ruckeri*** **(field isolates and reference strains) obtained using the combination of** ***Not*** **I and** ***Asc*** **I restriction enzymes and the BioNumerics software.** Origin/reference strains, host, biotype, serotype, pulsotype, number and region of origin are indicated. Clusters and subclusters are presented.

PFGE was performed on all 127 clinical isolates and the eight reference strains. It was considered appropriate to interpret the PFGE patterns observed for each restriction enzyme using the Tenover criteria for genetic analysis, more than ten bands having been obtained for the restriction profiles [25] (Figures 1, 2 and 3). For each dendrogram representation, only one representative of each pulsotype was included, except when the same pulsotype was found for several reference strains or for a reference strain and a field isolate. The number of isolates sharing the same pulsotype is indicated for each geographical area (Figures 1, 2 and 3).

After *Not*I digestion, 23 pulsotypes were recovered from the 127 clinical isolates and seven from the eight reference strains. One cluster (≥80.0% similarity) and one singleton were identified by analysis based on the Tenover criteria [25]. Specifically, there was one large cluster, C_N1_ with a similarity >80.3%, containing 126 clinical isolates and all reference strains. The singleton (pulsotype PtN29) corresponded to one isolate from a rainbow trout in Brittany and presented 66.0% of similarity with C_N1_ cluster (Figure 1). Three subclusters (≥90.0% similarity) were also obtained. Subcluster C_N1-Sc1_ was the most representative of the population with 114 clinical isolates and three reference strains, all the isolates in this cluster coming from rainbow trout. In this subtype (C_N1-Sc1_), two major pulsotypes were represented: PtN4 with 36 isolates, of which 29 came from Adour Garonne; and PtN7 with 36 isolates, of which 31 were from Brittany. The three reference strains present in this subcluster were: NCTC 12266, CIP 82.80^T^ and TUTI-EX5. The first of these, NCTC 12266 shared complete identity with two clinical isolates from Adour Garonne fish farms (pulsotype PtN6). The two other subclusters were represented by two or three distinct pulsotypes. C_N1-Sc2_ contained two isolates, one reference strain (NCTC 12268, PtN19) and one clinical isolate from sturgeon (PtN18), while C_N1-Sc3_ contained three clinical isolates from rainbow trout in Brittany (PtN21, 22 and 23). Outside these three subclusters, seven singletons were identified when similarity was >90.0%, of which four were clinical isolates (PtN20, 24, 25 and 28) and four reference strains (PtN1, 26 and 27). The mean D value obtained for the whole strain collection was 0.82 with significant variation according to the geographical area with values of 0.64, 0.68 and 0.84 for Brittany, Adour Garonne and other regions respectively (Table 1).

**Table 1:** Index of diversity (D) obtained for *Y. ruckeri* isolates from different geographical areas by the PFGE method with *Not*I or *Asc*I enzymes and their combination (*Not*I/*Asc*I)

	**Brittany**	**Adour Garonne**	**Other regions**	**All regions**
D *– Not*I	0.64	0.68	0.84	0.82
D *– Asc*I	0.49	0.80	0.63	0.70
D *– Not*I/*Asc*I	0.75	0.93	0.90	0.93

After *Asc*I digestion, of the same strain collection, 25 pulsotypes were obtained, of which 18 were from clinical isolates and seven from reference strains (Figure 2). For the same degree of clustering (similarity ≥80.0%), the digestion with *Asc*I enzyme indicated greater variability than that observed with *Not*I digestion. Indeed, four clusters were identified. One major cluster, C_A1_, contained 117 clinical isolates (92.1% of the collection) and three reference strains, CIP 82.80^T^, NCTC 12266 and TUTI-EX5. Clusters C_A2_ and C_A3_ included nine and one clinical isolates respectively. The C_A3_ cluster also included two reference strains (NCTC 12270 and NCTC 12269) and C_A4_ was represented by three reference strains (CECT 956, NCTC 12267 and NCTC 12268). For the whole strain collection, four subclusters were differentiated with a similarity ≥90.0%. One, C_A1-Sc1_, contained the great majority of the clinical isolates (*n* = 114; 89.7%). All clinical isolates in this cluster come from rainbow trout. The type strain CIP 82.80^T^ (PtA7) was the unique reference strain included in C_A1-Sc1._ The PtA1 and PtA5 pulsotypes dominated with 21 and 65 isolates respectively, PtA5 representing 51.2% of our strain collection with 37 isolates from Brittany, 21 from Adour Garonne and seven from other regions. The subcluster C_A1-Sc2_ contained two reference strains, NCTC 12266 (PtA13) and TUTI-EX5 (PtA14), C_A2-Sc1_ four clinical isolates (PtA17 and PtA18) and C_A3-SC1_ one clinical isolate from sturgeon (PtA21) and one reference strain, NCTC 12270 (PtA22). The mean D of the whole strain collection with the *Asc*I enzyme was 0.70, ranging between 0.49 and 0.80 depending of the geographical area observed (Table 1).

Combination of the two experiments (*Not*I and *Asc*I) based on a similarity matrix recovered 43 pulsotypes for the field isolates and seven for the reference strains (Figure 3). Two clusters were identified, a first one (C_N-A1_) containing six distinct pulsotypes representing five reference strains and two clinical isolates from gudgeon (PtN-A2) and sturgeon (PtN-A4), and a second one (C_N-A2_), representing the majority (98.4%) of the strain collection, including all rainbow trout clinical isolates, two sturgeon isolates and three reference strains (NCTC 12266, CIP 82.80^T^ and TUTI-EX5). Using a similarity ≥90.0%, two subclusters and ten singletons were obtained. The subcluster C_N-A2Sc1_ comprised only rainbow trout isolates and represented 117 isolates (92.1% of the strain collection). One pulsotype was dominant: PtN-A8 with 28 isolates of which 25 were from Brittany. Considering the whole strain collection, the maximum diversity observed was 79.0% (Figure 3). The D measured for our total collection was 0.93 (Table 1).

For the three experiments, *Not*I, *Asc*I and combined *Not*I/*Asc*I, PFGE did not enable us to discriminate biotypes one and two since the same pulsotype could be associated to either biotype (Figures 1, 2 and 3).

### Multi locus sequence typing

MLST was performed on the eight reference strains and 42 field isolates. One representative strain of each pulsotype identified by *Not*I or *Asc*I digestion was chosen in order to take into account the genetic diversity previously revealed by PFGE experiments (Table 2). The genetic characteristics of each locus are described in Table 3. Ten polymorphic sites were observed among the 50 strains studied, nine of which found among the field isolates only. No insertion or deletion was detected. The diversity index (H) calculated using the Lian software ranged from 0.0000 for *recA* gene to 0.4936 for *hsp60* gene. Pairwise nucleotide diversity (Pi) ranged from 0.0000 for *thrA* and *recA* to 0.00066 for *dnaJ*. The ratio of non-synonymous to synonymous mutation (dN/dS) was equal to 0.0000 or not determined for all loci, except for *dnaJ* gene for which dN/dS was to 0.2753 (Table 3).

**Table 2:** PFGE pulsotypes and MLST allele types and sequence types of 42 field isolates and 8 reference strains of *Y. ruckeri*

**Isolate/reference strain**	**Year/Reference**	**Region**	**Host**	**Biot.**	**Serot.**	**Pulsotype**			**Allelic profile**						**ST**
						**Pt N**	**Pt A**	**Pt N-A**	***glnA***	***gyrB***	***dnaJ***	***thrA***	***HSP60***	***recA***	
*TUTI EX5*	[48]	*UK*	*RT*	*2*	*O1*	*14*	*14*	*44*	*1*	*1*	*1*	*5*	*1*	*5*	*31*
*NCTC 12266*	[49]	*USA*	*RT*	*1*	*O1*	*6*	*13*	*34*	*1*	*1*	*1*	*5*	*1*	*5*	*31*
*CIP 82.80* ^*T*^	[49]	*USA*	*RT*	*1*	*O1*	*13*	*7*	*42*	*1*	*1*	*1*	*5*	*1*	*5*	*31*
CAE 224	2006	AG	RT	1	O1	4	18	15	1	1	1	5	1	5	31
CAE 709	2007	AG	RT	1	O1	4	10	40	1	1	1	5	1	5	31
CAE 710	2007	AG	RT	1	O1	3	11	41	1	1	1	5	1	5	31
CAE 358	2008	AG	RT	1	O1	6	6	32	1	1	1	5	1	5	31
CAE 361	2008	AG	RT	2	O1	16	5	23	1	1	1	5	1	5	31
CAE 376	2008	AG	RT	1	O1	4	1	10	1	1	1	5	1	5	31
CAE 425	2008	AG	RT	2	O1	20	5	7	1	1	1	5	1	5	31
CAE 642	2009	AG	RT	2	O1	4	12	14	1	1	1	5	1	5	31
CAE 700	2009	AG	RT	2	O1	4	9	39	1	1	1	5	1	5	31
CAE 385	2006	Brittany	RT	1	O1	8	5	28	1	1	1	5	1	5	31
CAE 393	2006	Brittany	RT	2	O1	24	5	9	1	1	1	5	1	5	31
CAE 396	2007	Brittany	RT	1	O1	24	17	48	1	1	1	5	1	5	31
CAE 399	2007	Brittany	RT	2	O1	5	5	8	1	1	1	5	1	5	31
CAE 406	2008	Brittany	RT	1	O1	5	5	8	1	1	1	5	1	5	31
CAE 410	2008	Brittany	RT	2	O1	7	5	8	1	1	1	5	1	5	31
CAE 657	2008	Brittany	RT	2	O1	7	5	8	1	1	1	5	1	5	31
CAE 534	2009	Brittany	RT	2	O1	21	2	45	1	1	1	5	1	5	31
CAE 535	2009	Brittany	RT	2	O1	8	8	36	1	1	1	5	1	5	31
CAE 536	2009	Brittany	RT	1	O1	23	2	47	1	1	1	5	1	5	31
CAE 538	2009	Brittany	RT	2	O1	22	2	46	1	1	1	5	1	5	31
CAE 684	2009	Brittany	RT	1	O1	7	1	22	1	1	1	5	1	5	31
CAE 436	2008	Other	RT	1	O1	12	1	12	1	1	1	5	1	5	31
CAE 678	2009	Other	RT	1	O1	7	1	22	1	1	1	5	1	5	31
*NCTC 12267*	[49]	*USA*	*Chinook Salmon*	*1*	*O2*	*27*	*24*	*1*	*5*	*1*	*2*	*5*	*1*	*5*	*32*
*CECT 956*	[49]	*USA*	*Chinook Salmon*	*1*	*O2*	*27*	*24*	*1*	*5*	*1*	*2*	*5*	*1*	*5*	*32*
*NCTC 12268*	[49]	*UK*	*RT*	*1*	*5*	*19*	*25*	*3*	*1*	*2*	*6*	*5*	*1*	*5*	*33*
*NCTC 12269*	[49]	*Canada*	*RT*	*1*	*O6*	*1*	*23*	*5*	*5*	*1*	*7*	*5*	*1*	*5*	*34*
*NCTC 12270*	[49]	*Denmark*	*Eel*	*1*	*O7*	*26*	*22*	*6*	*10*	*2*	*1*	*6*	*1*	*5*	*35*
CAE 212	2006	AG	RT	1	O1	4	16	43	1	1	1	5	5	5	36
CAE 220	2006	AG	RT	1	O1	4	20	17	1	1	1	5	5	5	36
CAE 711	2007	AG	RT	2	O5	17	8	37	1	1	1	5	5	5	36
CAE 363	2008	AG	RT	1	O1	10	5	18	1	1	1	5	5	5	36
CAE 367	2008	AG	RT	2	O1	10	3	19	1	1	1	5	5	5	36
CAE 370	2008	AG	RT	1	O1	11	5	20	1	1	1	5	5	5	36
CAE 428	2008	AG	RT	2	O1	17	5	7	1	1	1	5	5	5	36
CAE 645	2009	AG	RT	1	O1	2	12	14	1	1	1	5	5	5	36
CAE 209	2006	Brittany	RT	2	O1	10	5	28	1	1	1	5	5	5	36
CAE 397	2007	Brittany	RT	1	O1	9	5	29	1	1	1	5	5	5	36
CAE 658	2008	Brittany	RT	2	O1	4	4	11	1	1	1	5	5	5	36
CAE 660	2009	Brittany	RT	1	O1	29	5	50	1	1	1	5	5	5	36
CAE 666	2009	Brittany	RT	2	O1	4	1	10	1	1	1	5	5	5	36
CAE 675	2009	Brittany	RT	2	O1	12	1	26	1	1	1	5	5	5	36
CAE 676	2009	Brittany	RT	2	O1	7	1	22	1	1	1	5	5	5	36
CAE 362	2008	Other	RT	2	O1	15	5	25	1	1	1	5	5	5	36
CAE 431	2007	Other	Sturgeon	1	O6	28	15	49	1	1	6	5	5	5	37
CAE 432	2008	Other	Gudgeon	1	O1	25	19	2	7	2	4	6	1	5	38
CAE 713	2005	AG	Sturgeon	1	O1	18	21	4	1	2	2	5	1	5	39

Geographic origins are indicated by country or region for field isolates. Biot., biotype; Serot., serotype; Pt., pulsotype; AG., Adour Garonne; RT., Rainbow Trout. Reference strains are presented in italic format.

**Table 3:** Genetic characteristics of MLST loci

**Gene**	**Size (pb)**	**Mean GC (%)**	**Number of alleles**		**Number of polymorphic sites**		**Pi**		**H**		**dN/dS**	
			field isolates	field isolates + ref. strains	field isolates	field isolates + ref. strains	field isolates	field isolates + ref. strains	field isolates	field isolates + ref. strains	field isolates	field isolates + ref. strains
*glnA*	416	50.48	2	4	1	2	0.0003	0.0007	0.0476	0.1894	0.0000	0.0000
*gyrB*	454	45.59	2	2	2	2	0.00011	0.00055	0.0929	0.1502	0.0000	0.0000
*dnaJ*	632	53.64	4	5	4	4	0.00041	0.00066	0.1394	0.2596	0.2753	0.2753
*thrA*	303	47.85	2	2	1	1	0.0000	0.0000	0.0476	0.0784	-	-
*hsp60*	509	47.74	2	2	1	1	0.00016	0.00026	0.4936	0.4580	-	-
*recA*	472	49.58	1	1	0	0	0.0000	0.0000	0.0000	0.0000	-	-

- Not determined.

The MLST identified nine STs among the 50 strains. The rainbow trout field isolates were represented by two STs, ST31 (59%) and ST36 (41%), isolates from sturgeon were identified as ST37 and ST39 and the only gudgeon isolate as ST38 (Table 2). Five different STs were found among the eight reference strains, STs 31, 32, 33, 34 and 35 (Table 2). These results showed a low genetic diversity for the outbreak isolates with a diversity index of 0.62 for the whole population studied.

**Figure 4:**
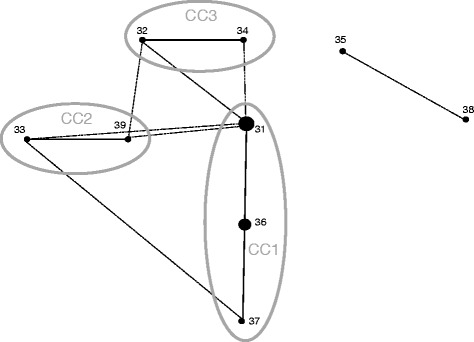
**e-BURST diagram. Single locus variants are joined by straight lines and double locus variants by dotted lines.** Clonal complexes (CC) were represented by a circle. Dot sizes represent the number of isolates within each ST.

The e-BURST method gives information about the predicted evolution of isolates (Figure 4). With default parameters, STs with five common ATs were linked and grouped into a clonal complex (CC). One clonal complex (CC1) with STs 36, 37 and 31 was described, representing 44 isolates and ST 36 was defined as the predicted founder. Two others clonal complexes were described, CC2 with ST 33 and ST 39 and CC3 with ST 32 and ST 34. ST 38 and ST 35 were defined as singletons (Figure 4). With the relaxed parameters, STs with four ATs were linked, connections between clonal complex one, two and three were observed and also between the two singletons (Figure 4).

The standardized index of association, I^S^_A_, was calculated for (i) all 50 strains; (ii) the 42 field isolates; (iii) the dataset comprising a single representatives of the ST; and (iv) the dataset comprising a single field isolate representatives of the 5 ST (Table 4). For the first two groups of strains I^S^_A_ values were respectively 0.1655 and 0.2022 and were significantly different from 0 (*P* < 0.05) indicating a linkage disequilibrium (Table 4). This linkage disequilibrium disappears for the two last groups of strains studied, with I^S^_A_ values close to 0 (*P* > 0.05) (Table 4).

**Table 4:** Analysis of linkage disequilibrium in MLST data by START2 software

**Group (n)**	**Ve**	**Vo**	**I** ^**S**^_**A**_	***P***
Total isolates (50)	0.7938	1.4506	0.1655	0.000*
Field isolates (42)	0.5449	1.0959	0.2022	0.000*
Total STs (9)	0.8418	0.7897	−0.0124	0.706
Total Field STs (5)	0.81	1.1667	0.0881	0.324

*Significant linkage disequilibrum.

Ve, Values for expected variance.

Vo, values for observed variance.

I^S^_A_, Standardized index association.

*P*, Values.

**Figure 5:**
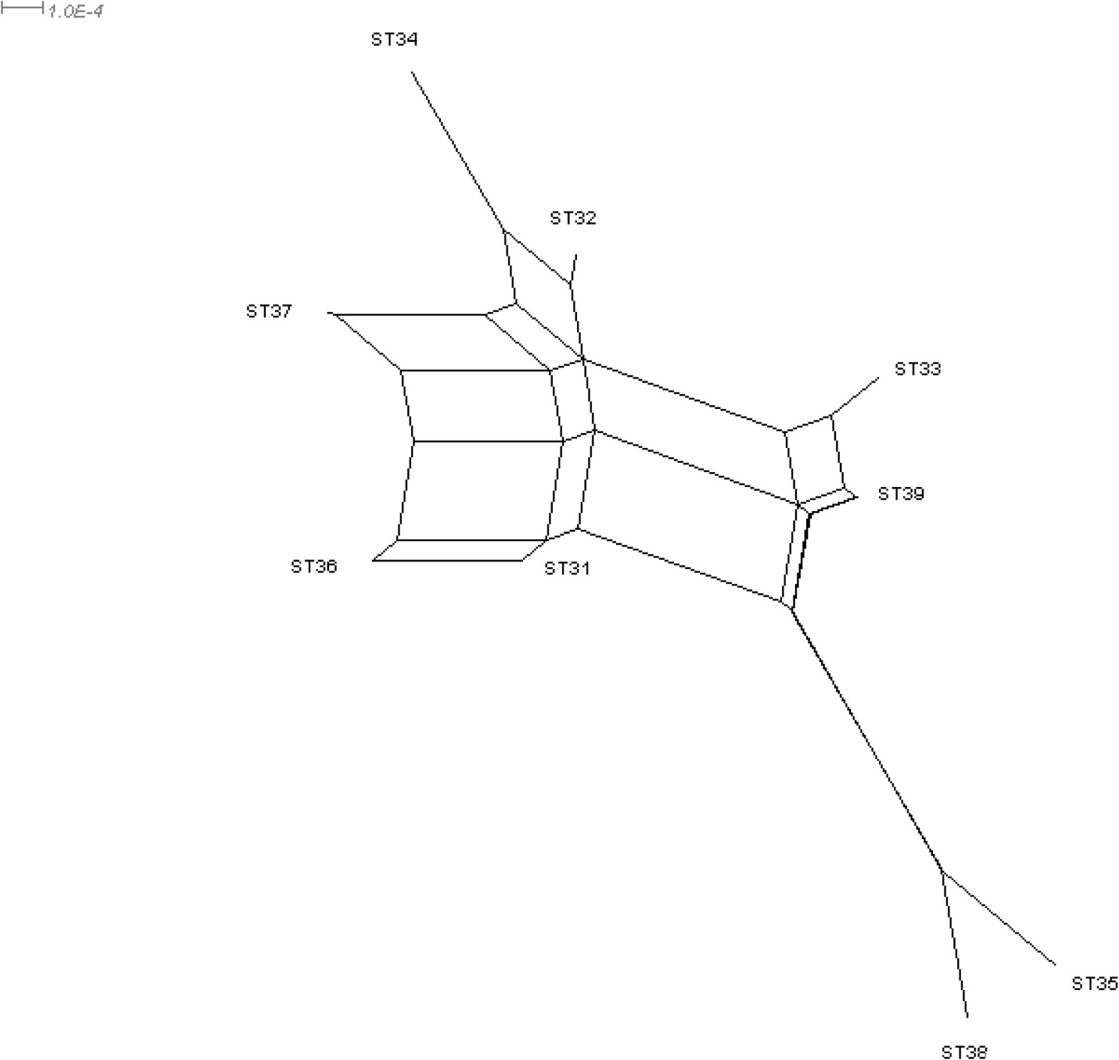
**Split decomposition analysis with the concatenated sequences of each ST.** The concatenated ST sequences showed a connecting network structure.

The pairwise homoplasy index (PHI) was obtained only for *gyrB* and *dnaJ* loci and was equal to 1, representing no significant recombination event (*p* > 0.05). The PHI value for the concatenated sequences was 0.14. The Splits Tree program was used on these concatenated ST sequences and showed a connecting network structure suggesting recombination (Figure 5).

## Discussion

*Y. ruckeri* is an important bacterial pathogen in aquaculture worldwide. Its presence on fish farms requires close surveillance and the use of vaccination and antimicrobial agents. Analysis of its genetic diversity and population structure using PFGE and MLST is required to improve our understanding of this pathogen and, in turn, farm management. In our PFGE analysis, two levels of diversity were identified: one general level, indicating a low overall diversity of the French clinical *Y. ruckeri* population with 2 to 4 clusters (≥80.0%) depending on the enzyme used (*Not*I, *Asc*I or *Not*I/*Asc*I); and a second level, finer level, indicating high diversity within the individual clusters C_N1_ and C_N-A2_ representing 99.2% and 98.4% of the clinical population and 28 to 43 different pulsotypes, respectively. Also using PFGE, Lucangeli et al., described one profile for 30 Italian isolates from rainbow trout [17], Ström-Bestor et al., 19 pulsotypes for 52 Northern European isolates [34] and Huang et al., 17 pulsotypes in three clusters for 83 German isolates [35]. Another study based on strains collected worldwide over a 30-year period from many geographical origins and different hosts found a similar clonal structure [18]. Specifically, in that study in 2009, Wheeler et al. [18] assigned 160 isolates to 44 pulsotypes with a minimum of 70.0% of similarity and a very similar group, Pt31 to Pt39, represented most of the population. Pulsotype Pt35 represented 45/56 of European rainbow trout isolates, the exceptions being those from the United Kingdom. Overall, the results indicated low diversity and were in accordance with a clonal population structure. Comparison of our results with those of the three other studies in which the *Not*I enzyme was used in PFGE [18,34,35] reveals great similarities. Indeed, despite differences in the number and geographical origin of the isolates, the minimum degrees of homology were similar, i.e. 85.0%, 70.0% and 63.6% in the studies by Ström-Bestor et al. [34], Wheeler et al. [18] and Huang et al. [35] respectively and 66.0% in our sample. A low genetic diversity is often observed in animal and human bacterial pathogens, such as *Yersinia pestis* and *Mycobacterium tuberculosis* [36], and this could be associated with their narrow niches, particularly eukaryotic host cells [37], fish in the case of *Y. ruckeri*.

On the other hand, the high general homology associated with a slow rate of evolution is independent of the considerable diversity observed by PFGE, with D = 0.79 in one publication [35] and D = 0.82 in our work (Table 1). A visual comparison permits matching of the main profiles in the four studies: Pt35 [18] = pf1 [34] = C1 [35] = PtN4 (our work), PtN4 corresponding to Adour Garonne isolates; and Pt37 [18] = C10 [35] = PtN7 (our work), PtN7 corresponding to Brittany isolates (Figure 1). These observations are all in accordance with a clonal population structure. Other studies based on different techniques, such as RFLP [14] and multilocus enzyme electrophoresis [38] have identified the same clonal structure population for *Y. ruckeri*.

Recently, other authors using MLST showed the persistence of two major sequence types (ST1 and ST2) and one clonal complex (CC1) over a period of 30 years in a collection of 103 isolates from different geographical areas and hosts [19]. Their hypothesis was an epidemic model of clonal expansion for *Y. ruckeri* as described for another bacterial fish pathogen, *Flavobacterium psychrophilum* [39,40] and other bacterial species such as *Pseudomonas aeruginosa* [41]. Our results, in a limited geographical area reached the same conclusion, i.e. an epidemic model for *Y. ruckeri* population when I^S^_A_ was performed on single representatives of each STs (Table 4). Only ST 31 and ST 36 were represented among the French rainbow trout isolates. These STs differ by only one base in N terminal position, the probable consequence of a mutation event. The PHI values obtained for loci seem to indicate no role of recombination in population evolution. The hypothesis of a clonal population for the field French isolates was dominant by using the MLST approach.

No common ST was obtained in our and Bastardo’s studies because differences were observed in the *recA* and *thrA* gene sequences between the different batches of the type strains used in the two studies, i.e. CIP 82.80^T^ and NCIMB 2194^T^, respectively. We found that the sequence of strain CIP 82.80^T^ used in our study is identical to that of a third batch of the type strain, ATCC 29473^T^ (NCBI GCA_000173755.1). The sequence differences observed between strains CIP 82.80^T^ and NCIMB 2194^T^ may be explained by the rapid adaptation of bacteria to in vitro conditions [42]. To compare the studies, we considered the strains CIP 82.80^T^ and NCIMB 2194^T^ as similar and corresponding to the ST1 as described by Bastardo et al. [19]. In this way another ST was common between the two populations studied, the ST38 = ST22 [19]. The e-BURST constructed with these new data show ST 14 as a predicted founder as previously described by Bastardo et al. [19], while STs 33, 36, 37 and 39 were comprised in CC1, STs 32 and 34 in a third group and ST35 was described as a singleton (data not shown).

Comparison of the DNA sequences of several batches of the same strain may reveal changes caused by preservation conditions, frequency of culture, etc. In this study, PFGE showed that strains CECT 956 and NCTC 12267 shared the same three pulsotypes (PtN27 – PtA24 – PtN-A1), as well as the same serotype (O2), biotype (BT1) and source (Chinook salmon). Hence, it is highly likely that they actually represent the same strain deposited in two different culture collections. This confirms the ability of PFGE as an interesting method for tracing the origin of bacterial strains.

A clone was defined as a group of isolates expressing the same characteristic phenotype and genotype (PFGE pattern similarity >90.0%) [43]. However, in our study and others [18,35], many isolates presenting a pattern similarity >90.0% actually displayed a different phenotype in terms of motility. Hence, two isolates having the same pulsotype may actually not belong to the same clone.

In other studies, authors used the RFLP method to describe mutations involved in mobility and characterize the biotype 2 of isolates. Considering the different mutations observed in genes (fliR, flhA and flhB) depending on geographical origin of isolates analyzed, the authors suggest that the evolution of *Y. ruckeri* is different across Europe [44,45]. This independent evolution could be explained by fish culture practices, therapeutic and prophylactic strategies, and/or movements of farmed fish. These observations and the different levels of diversity observed using PFGE and MLST showed the importance of the choice of marker/method to analyse the diversity or population structure of a bacterial species.

The combination of typing methods could be an interesting approach for evaluating population diversity. Bastardo et al. using a polyphasic approach, combining biotyping, API20E galeries, OMP, LPS, ERIC-PCR and REP PCR on 71 *Y. ruckeri* isolates from different countries and hosts, showed a dominance of some subgroups in certain areas as well as host specificity and a very high diversity with a D of 0.90 [16]. In the present study, PFGE with *Not*I and *Asc*I combined yielded additional information and a greater discriminatory power with a D = 0.93 and 50 pulsotypes observed for the whole strain collection compared to D of 0.82 or 0.70 and 29 or 25 pulsotypes for *Not*I and *Asc*I used separately, respectively (Figures 1, 2, 3 and Table 1) or compared to the MLST approach with a D value of 0.62.

In our study, pulsotypes and STs seem to be associated with a host fish species as described in other PFGE [35] and MLST [19] studies. This association indicates adaptive niche specialization and some authors have suggested that alternative niche development has played greater importance in the dissemination and evolution of *Y. ruckeri* than preferential routes of transmission [19,45]. The fact that most isolates from different geographical origins have the same pulsotype (PtN7 for the West of France (Brittany) and C10 for West Germany, or PtN4 for the South West of France (Adour Garonne) and C1 for West Germany), reflects the importance of movements of farmed fish between regions more than an evolution dependent on geographical area (environment or practices). The different values of D between regions pointed to a greater population homogeneity for isolates from Brittany than Adour Garonne (Table 1). The different D values and the identical size of sample for these two areas (54 isolates each) suggest an influence of geographical origin. It seems that different selection pressures, specific ecological niches, movements of farmed fish, and geographical origins, all play a role in *Y. ruckeri* dissemination and evolution.

To obtain additional information on *Y. ruckeri* diversity and population structure and to compare the results of MLST and PFGE it would be interesting to apply these methods to isolates retrieved from a larger variety of geographical areas (Europe, or beyond) as described for other bacterial pathogens [46,47]. In addition analysis of environmental isolates compared to clinical isolates would be pertinent to explore differences between ecological niches and to identify any specific linkage that could explain the survival in the environment of pathogens and the recurrence of outbreaks under specific conditions (temperature, stress).

## Abbreviations

AG: Adour garonne
CC: Clonal complex
ERIC: Enterobacterial repetitive intergenic consensus
LPS: LipoPolySaccharide
MLST: Multi locus sequence typing
OMP: Outer membrane protein
PFGE: Pulsed-field gel electrophoresis
Pt: Pulsotype
REP: Repetitive extragenic palindromic
RT: Rainbow trout
ST: Sequence type
